# Editorial: Anti-tumor activity of cytotoxic immune cells: basic research and clinical perspectives

**DOI:** 10.3389/fimmu.2024.1428565

**Published:** 2024-05-15

**Authors:** Malgorzata Firczuk, Magdalena Winiarska

**Affiliations:** ^1^ Laboratory of Immunology, Mossakowski Medical Research Institute Polish Academy of Sciences, Warsaw, Poland; ^2^ Department of Immunology, Medical University of Warsaw, Warsaw, Poland

**Keywords:** cancer immunotherapy, T cell, NK cell, cytotoxic cell, CAR-T cell, monoclonal antibody, TME (tumor microenvironment), clinical trial

Immunotherapy has become an important strategy for the treatment of cancers. Currently available cancer immunotherapies include monoclonal antibodies, bispecific T cell engagers, tumor vaccines, T cells modified with chimeric antigen receptors (CAR-T), and immune checkpoint inhibitors (ICIs). In the vast majority of cases, current immunotherapy is based on the engagement of cytotoxic cells. This Research Topic explores T cell activity regulation and the use of cytotoxic T cell and NK cell-based methods in cancer treatment through a compilation of experimental and review articles. It covers studies on pathways governing T cell activation, the mechanisms orchestrating inter and intra-cellular communication among T cells and the tumor microenvironment (TME), and the prognostic significance of distinct T cell subpopulations across diverse cancer types. Additionally, the effective utilization of cytotoxic T cells and NK cells for cancer immunotherapy, specifically by leveraging monoclonal antibodies, ICIs, and CAR-T cells is discussed. Furthermore, insights derived from clinical trials evaluating the efficacy of cancer immunotherapies are presented. These contributions furnish important perspectives to the dynamic landscape of cancer treatment.

Three articles focus on the functional aspects of T cell activity. Firstly, Monticone et al. present an original paper introducing a novel immunosuppressive pathway within T cells. This pathway, involving adenosine receptor A2AR, Cbl-b, and Notch1, acts as a distinct immune checkpoint in the TME, modulating T cell responses. The authors demonstrate that enhancing Notch1 signaling, by impeding Cbl-b-mediated degradation, substantially boosts anti-cancer T cell responses, providing an innovative immunotherapeutic strategy with potential selectivity for T cells over cancer cells. The review article by Zhou et al. focuses on T cell-derived exosomes. It explores the dual effects of CD8 and CD4 T cell-derived exosomes on tumor progression, with insights into modifying exosome surfaces for therapeutic intervention. The role of regulatory T cells (Tregs)-derived exosomes in tumor immune escape is also discussed, suggesting novel cancer immunotherapy through targeting of Treg-derived exosomes. The review also points to engineered T cell-derived exosomes as a potential drug delivery system with high stability and low immunogenicity. Finally, the article by Moussawy et al. reviews the non-cytotoxic functions of CD8 T cells, including their role in cancer immunotherapy, in particular in anti-tumoral vaccination. Addressing weak immunogenicity of tumoral antigens, the article describes how bystander CD8 T cells enhance the anti-tumoral effect of dendritic cell-based vaccines, suggesting their role as potent adjuvants.

The two subsequent original papers focus on detecting specific tumor-infiltrating lymphocytes (TILs) subpopulations in certain types of cancer, exploring their prognostic functions and discussing their potential impact on immunotherapy efficacy. In Vlaming et al.‘s article, a rare CD8 T cell subpopulation expressing CD103/TIM-3/CXCL13 markers was found to be associated with improved survival of epithelial ovarian cancer patients. TIM-3/CD8/CD103-positive T cells can serve as a prognostic marker for epithelial ovarian cancer patients and as a target population for reactivation by immunotherapeutics. The article by Zhou et al. focuses on the quantitative evaluation of Tregs in nasopharyngeal cancer. Tregs play a crucial role in suppressing antitumor immunity in the TME. The study outlines the infiltrating profile and spatial distribution of TILs in nasopharyngeal cancer, examining the prognostic value of TILs composition, spatial architecture, and PDL1 expression on TILs subpopulations in a large cohort of nasopharyngeal cancer patients. Increased infiltration of Tregs, especially PDL1+ Tregs, near tumor cells and CTLs correlates with unfavorable outcomes, highlighting the crucial role of dynamic intercellular interactions between heterogeneous T cell subtypes in disease progression. The findings suggest that PDL1+ Tregs interact with CTLs via the PD1/PDL1 axis, mediating CTL dysfunction and enhancing immune suppression, with implications for future clinical investigations and immunotherapy in nasopharyngeal cancer patients.

Finally, four articles focus on harnessing cytotoxic T and NK cells for cancer immunotherapy. The review article by Dabkowska et al. presents advancements in cancer immunotherapies targeting CD20. It highlights the revolutionary impact of CD20-targeting immunotherapies like rituximab and further discusses novel advancements such as bispecific T cell engagers and CAR-T cells. The article focuses mainly on CD20-targeting immunotherapeutics that are clinically approved or tested in clinical trials. The article by Singh et al. presents promising safety results from a phase 1 clinical trial involving non-virally modified CAR-T cells, employing the sleeping beauty transposon-based approach. This innovative T cell modification strategy, characterized by shortened manufacturing time, demonstrates both safety and cost-effectiveness, offering promising antitumor activity, particularly beneficial in the context of solid tumor immunotherapy. The article by Tomasik et al. provides an in-depth overview of FDA-approved 2nd generation CAR-T cell products and explores the advancements in 3rd and next-generation CAR constructs, summarizing initial results of clinical trials. Although 3rd-generation CAR-T cells, incorporating two costimulatory domains in their CAR constructs, exhibit improved expansion and persistence, their response rates are similar to conventional CAR-T therapies. Ongoing clinical trials investigate various additional approaches, including immune checkpoint modulation, cytokine secretion, safety-switch mechanisms, and genetically edited CAR-T cells. Some of these innovative solutions demonstrate promising potential, achieving response rates of up to 100%, however, they still await evaluation in bigger patient cohorts. Additionally, CRISPR-KO technologies contribute to the development of off-the-shelf CAR-T cells by knocking out TCR and MHC molecules, offering cost and time-saving advantages. Ongoing trials will evaluate their efficacy, safety, and potential concerns, such as susceptibility to natural killer cell-mediated cytotoxicity. As the authors suggest, the strategic combination of efficacy enhancers with safety switches emerges as a rational approach, pending in-depth analysis and comprehensive trial results. Finally, a comprehensive review article by He and Hu focuses on the progress in utilizing ICIs for small-cell lung cancer. ICIs monotherapy and combination therapy have become established as standard options for small-cell lung cancer patients, with ongoing research aiming to further improve ICI immunotherapy by investigating novel combination strategies involving chemotherapeutics and radiation treatment. The article addresses current limitations and explores prospects for future developments, marking significant progress in both first- and third-line treatments for small-cell lung cancer patients.

In conclusion, the research outlined in these articles underscores the dynamic progress and potential avenues within cytotoxic cell-based cancer immunotherapies.

## Author contributions

MF: Conceptualization, Writing – original draft, Writing – review & editing. MW: Writing – review & editing.

